# Early Signal Without Clinical Cases: A Single Clade III *Candidozyma auris* Isolate from a Face Mask Highlights the Value of Environmental Quality Control

**DOI:** 10.3390/jof12050307

**Published:** 2026-04-23

**Authors:** Angelika Bauer, Astrid Mayr, Stephanie Toepfer, Kathrin Spettel, Birgit Willinger, Richard Kriz, Cornelia Lass-Flörl

**Affiliations:** 1 European Excellence Centre of Medical Mycology, Institute of Hygiene and Medical Microbiology, Medical University of Innsbruck, 6020 Innsbruck, Austria; astrid.mayr@i-med.ac.at (A.M.); stephanie.toepfer@i-med.ac.at (S.T.); cornelia.lass-floerl@i-med.ac.at (C.L.-F.); 2 Division of Clinical Microbiology, Department of Laboratory Medicine, Medical University of Vienna, 1090 Vienna, Austria; kathrin.spettel@meduniwien.ac.at (K.S.); birgit.willinger@meduniwien.ac.at (B.W.); richard.kriz@hcw.ac.at (R.K.); 3 Comprehensive Center for Infection Medicine, Medical University of Vienna, 1090 Vienna, Austria; 4 Section Biomedical Science, Health Sciences, University of Applied Sciences Campus Wien, 1100 Vienna, Austria

**Keywords:** *Candidozyma auris* (*Candida auris*), antifungal resistance, healthcare-associated infections, infection prevention and control

## Abstract

*Candidozyma auris* (*C. auris*) is an emerging healthcare-associated yeast of major epidemiological concern because of its multidrug resistance and outbreak potential. We report the recovery of a single *C. auris* isolate from a used face mask collected in May 2025 during a blinded dental medicine quality-control programme assessing microbial contamination in the working environment. To contextualise this finding, we analysed routine diagnostic laboratory data from 2017 to 2025. The isolate underwent whole-genome sequencing for molecular characterisation, including analysis of the *ERG11* gene, and antifungal susceptibility testing by EUCAST broth microdilution. In addition, 53,802 patient-related *Candida* spp. isolates collected between 2017 and 2025 were reviewed retrospectively; species identification had been performed by MALDI-TOF. The environmental isolate belonged to clade III and carried the V125A/F126L substitutions in *ERG11*, consistent with African clade isolates and associated with intrinsically high fluconazole minimum inhibitory concentrations. No *C. auris* was detected in routine patient specimens during the study period, whereas *Candida albicans* remained the predominant species in clinical samples. These findings provide no evidence of ongoing *C. auris* transmission at the Medical University of Innsbruck, but highlight the need for continued vigilance and robust infection-prevention measures to limit the risk posed by isolated introductions.

## 1. Introduction

**Figure 1:**
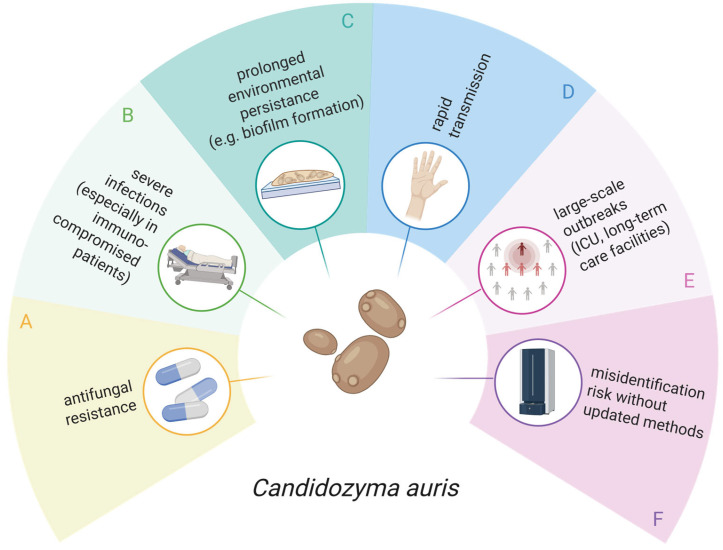
Pathogenicity and clinical significance of *Candidozyma auris*. Schematic overview illustrating why *C. auris* is an emerging fungal pathogen of global concern. (**A**) Many *C. auris* isolates show reduced susceptibility or resistance to antifungal agents, most commonly to azoles, followed by amphotericin B, and only rarely to echinocandins. (**B**) *C. auris*-associated candidaemia and invasive candidiasis are associated with fatality rates up to 60%, particularly in immunocompromised and other high-risk patients. (**C**) Owing to its ability to form multilayer biofilms, *C. auris* can persist in challenging environments, including high-salt and high-temperature conditions, and on medical devices. (**D**) It can also be transmitted easily, particularly via contaminated surfaces, such as medical equipment or through skin contact with asymptomatic carriers. (**E**) These characteristics facilitate large-scale outbreaks, which often occur in intensive care units (ICUs) and long-term care facilities. (**F**) Because *C. auris* is closely related to *Candida haemulonii* and *Candida duobushaemulonii*, accurate species-level identification requires reliable methods such as matrix-assisted laser desorption time-of-flight mass spectrometry with updated databases, sequencing, PCR or loop-mediated isothermal amplification [1,4]. Created in BioRender; Bauer, A. https://BioRender.com/igvq15p (2026).

*Candidozyma auris* (*Candida auris*) has emerged as a healthcare-associated yeast of major concern because of its persistent colonisation of human skin and medical devices, frequent multidrug resistance, and strong association with difficult-to-control outbreaks in healthcare settings, particularly among immunocompromised patients with severe infections (Figure 1) [1]. Clinical manifestations range from candidaemia to device-associated infections, as well as wound and soft tissue, urinary tract, and ear infections, depending on the patient population. Importantly, asymptomatic carriage with prolonged skin colonisation, predominantly involving the nares, axilla, and groin, is common. This represents a major risk factor for transmission and subsequent invasive disease [2,3].

## 2. Materials and Methods

A *C. auris* isolate (internal laboratory ID: CAU18) was recovered in May 2025 from a used face mask worn by a dental student during clinical activities at the Medical University of Innsbruck. The isolate originated from a blinded, anonymised quality-control programme assessing microbial contamination in dental working environments. Thirty used face masks collected in the dental clinic were screened for the presence of *Candida* spp.

Detection was performed by contact sampling on CHROMagar^TM^ Candida Plus agar (Mast Diagnostica, Reinfeld, Germany). Species-level identification was subsequently confirmed by matrix-assisted laser desorption time-of-flight mass-spectrometry (MALDI-TOF MS), as described below.

All patient-related *Candida* spp. isolates analysed at the Institute of Hygiene and Medical Microbiology, Medical University of Innsbruck, between 2017 and 2025 were included retrospectively. Collected data comprised diagnostic findings and patient sex as a demographic variable. Samples were categorised as invasive, non-invasive, or of unknown origin. Invasive samples underwent species-level identification in almost all cases, whereas non-invasive samples were identified to the species level less consistently in routine diagnostics. In routine laboratory practice, samples that did not undergo species-level identification were reported as *Candida* spp. based on microscopy of suspected colonies grown on Sabouraud agar or Columbia blood agar, or recovered from Sabouraud broth. These findings were always supported by microscopy of the original specimen. Where microscopy was inconclusive, samples were cultured on CHROMID^®^ *Candida* agar (bioMérieux, Marcy-l’Etoile, France) to enable presumptive differentiation by colony colour. MALDI-TOF MS was then used to support identification.

Candida species identification

Samples positive for *Candida* spp. were cultured on CHROMID^®^ *Candida* agar to enable preliminary differentiation based on colony colour. Definitive species identification was then performed by MALDI-TOF MS using the MALDI Biotyper Smart system with the Biotyper library v.4.1 (Bruker Daltonik GmbH, Bremen, Germany). Identification scores of ≥2.0 were interpreted as reliable for species-level identification.

Whole-genome sequencing (WGS)-based phylogeographic clade assignment

For rapid clade assignment, an allele-specific PCR for *C. auris* [5] was performed at the Medical University of Innsbruck. Genomic DNA was extracted using the Yeast DNA Extraction Kit (Thermo Fisher Scientific, Waltham, MA, USA) according to the manufacturer’s instructions, and PCR was performed as described previously [5]. In addition, the *ERG11* gene was sequenced to identify clade-associated mutations. To confirm these findings further, WGS was performed at the Austrian National Reference Centre for Yeasts and Moulds (Vienna), as previously reported [6]. WGS data for the *C. auris* isolate CAU18 are available under NCBI BioSample SAMN55402253.

Antifungal susceptibility testing

Antifungal susceptibility testing was performed by broth microdilution in accordance with the recommendations of the European Committee on Antimicrobial Susceptibility Testing [7]. Minimum inhibitory concentrations (MICs) were determined for the following antifungal agents at the indicated concentration ranges: anidulafungin (0.008–16 mg/L), micafungin (0.008–16 mg/L), caspofungin (0.008–16 mg/L), fluconazole (0.125–256 mg/L), posaconazole (0.016–32 mg/L), voriconazole (0.008–16 mg/L), 5-flucytosine (0.032–64 mg/L), amphotericin B (0.032–16 mg/L), and manogepix (0.002–16 mg/L).

## 3. Results

### 3.1. Environmental Detection and Characterisation of the C. auris Isolate

In May 2025, a single *C. auris* isolate was recovered from a used face mask worn by a dental student during clinical activities in Innsbruck, Austria. The sample originated from a structured environmental quality-control programme designed to assess microbial contamination in the dental working environment. Of the thirty used masks evaluated, one (3.3%) tested positive for *C. auris*, five (16.7%) for *C. albicans*, and five (16.7%) for *C. parapsilosis*. No other *Candida* species were detected. The program was not performed in response to a suspected outbreak or a known *C. auris* case. Because the programme was conducted in a blinded and anonymised manner, the wearer could not be identified retrospectively. Consequently, no follow-up sampling, including colonisation screening, could be performed, and no exposure history, such as recent travel or prior healthcare contact, could be obtained.

The isolate was identified as *C. auris* and assigned to clade III. Sequence analysis of the *ERG11* gene revealed the V125A/F126L amino acid substitutions previously associated with azole resistance [8]. The isolate demonstrated a high MIC to fluconazole (256 mg/L), whereas MICs for voriconazole and posaconazole were lower at 1 mg/L and 0.25 mg/L, respectively. MICs for the echinocandins were low (anidulafungin 0.25 mg/L, micafungin 0.125 mg/L, caspofungin 0.25 mg/L). The MIC was 2 mg/L for amphotericin B, 0.125 mg/L for flucytosine, and 0.008 mg/L for manogepix.

At the time of the investigation, no additional *C. auris* isolates were recovered from environmental samples collected by the hospital hygiene team. This argues against widespread environmental contamination during that period.

### 3.2. Local Laboratory Observations and Intensified Yeast Species Identification (2017–2025)

**Figure 2:**
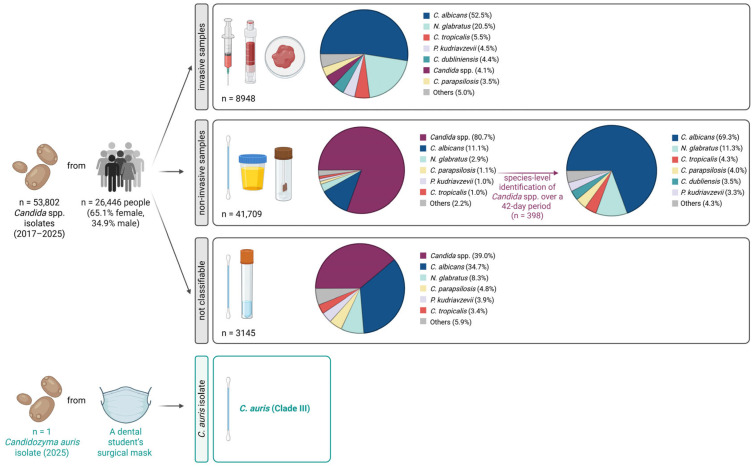
Overview of clinical samples positive for *Candida* spp. analysed between 2017 and 2025 (n = 53,802). Samples were categorised as invasive, non-invasive, or of unknown clinical relevance where classification was not possible. Species-level identification was performed by culture on CHROMID^®^ *Candida* agar followed by matrix-assisted laser desorption time-of-flight mass-spectrometry, and the results are summarised in the pie charts. In addition, a single *Candidozyma auris* (*C. auris*) isolate was recovered from a used face mask and was assigned to clade III. Created in BioRender; Bauer, A. https://BioRender.com/2kiu68e (2026).

To provide longitudinal context around the first documented human *C. auris* case in Austria in 2018 [6], we retrospectively reviewed all *Candida* spp. detections recorded in our routine diagnostic laboratory from 2017 onwards. Over the 9-year study period (2017–2025), 8948 invasive specimens and 41,709 non-invasive specimens were processed for yeast growth as part of routine clinical microbiology diagnostics (Figure 2, Table 1). In addition, 3145 specimens of unknown origin were included. Overall, 53,802 specimens from 26,446 individuals were analysed; 65.1% of individuals were female, largely because of the high number of vaginal swab submissions.

Across the study period, *C. albicans* was the most frequently detected species, accounting for 54.7% of invasive samples and 57.4% of non-invasive samples that underwent species-level identification. This was followed by *Nakaseomyces glabratus*, identified in 21.3% of invasive samples and 15.2% of non-invasive samples. In routine practice, species-level identification was performed for nearly all invasive specimens, whereas non-invasive and specimens of unknown origin were usually reported as *Candida* spp. unless further identification was specifically requested. Consequently, only a small portion of non-invasive samples underwent species-level identification. Because this approach could theoretically have missed sporadic *C. auris* detections in non-invasive material, species-level identification was intensified after the environmental finding. During an additional 42-day period, 398 yeast-positive non-invasive specimens were further analysed by CHROMID^®^ *Candida* agar and MALDI-TOF MS. No non-classifiable samples were submitted to the routine laboratory during this period. Accordingly, intensified diagnostics were limited to non-invasive specimens. This was likely due to improvements in pre-analytical procedures over the years, including a revised laboratory request form completed by the submitting clinicians or institutions and mandatory follow-up calls by the routine microbiology laboratory when non-classifiable samples are received. This short-term analysis showed a similar species distribution, with *C. albicans* remaining the dominant species and accounting for 69.3% of non-invasive samples. No *C. auris* was detected in any clinical specimens during the observation period.

## 4. Discussion

Interpretation of a single mask-positive finding in the absence of clinical detections

The observation of a single mask-positive isolate in the absence of positive routine clinical specimens may be explained by several non-mutually exclusive factors. However, these explanations remain speculative and should be regarded as hypothesis-generating only, as the study was based on a single environmental isolate and the anonymised design precluded epidemiological follow-up. Importantly, this finding does not provide evidence of transmission or the presence of *C. auris* in the underlying population.

First, routine diagnostics should not be equated with colonisation screening. In our setting, the laboratory primarily processes blood cultures, urine, respiratory specimens, and wound swabs. The absence of *C. auris* in these materials does not exclude unrecognised asymptomatic carriage, particularly at typical colonisation sites that are not routinely screened but are central to transmission [2,3].

Second, the source of the mask-associated isolate remains uncertain. It could not be determined retrospectively whether the isolate originated from the inner or outer surface of the mask. In addition, the anonymised quality-control design prevented re-identification of the wearer and confirmatory follow-up. The finding may therefore reflect nasal colonisation; transient hand-to-mask transfer after contact with contaminated surfaces, equipment, or gloves; or contamination of the outer mask surface within the clinical environment. This interpretation is compatible with the prolonged viability of *C. auris* under dry conditions [2]. Nonetheless, because colonisation cannot be distinguished from contamination, any further interpretation remains speculative.

Third, the isolate belonged to clade III, which has been reported in multiple regions worldwide, including South Africa, the United Kingdom, China, Saudi Arabia, Spain, Australia, Canada, Germany, and Austria [4,6]. However, possible routes of introduction could not be assessed because of the blinded and anonymised sampling design. Moreover, the *C. auris* isolate showed no close genetic relationship to any previously detected *C. auris* isolate in Austria [6].

Implications and conclusion

Although no *C. auris* outbreak was detected at the Medical University of Innsbruck during the study period, even an isolated detection warrants attention, as sporadic introductions may precede transmission under favourable conditions. The fact that the isolate was identified through a routine, blinded environmental quality control programme, rather than a case-driven investigation, highlights the potential value of such monitoring for the early detection of unusual or emerging pathogens in healthcare-associated settings, including dental medicine. Given the well-documented ability of *C. auris* to persist on dry, frequently touched surfaces, consistent environmental cleaning with agents active against this pathogen remains essential. Additionally, strict adherence to hand hygiene must complement the environmental cleaning and disinfection measures [9].

## 5. Conclusions

In summary, we recovered a single clade III *C. auris* isolate from a used face mask collected during a dental environmental quality-control programme in May 2025. By contrast, *C. auris* was not detected in routine patient specimens processed between 2017 and 2025, including during an intensified 42-day period of expanded species-level identification. Although these findings are consistent with the absence of recognised clinical transmission in our setting, the lack of systematic colonisation screening and the anonymised sampling design limit conclusions regarding silent carriage and the route of introduction. Continued vigilance and laboratory preparedness therefore remain essential, as even a single introduction may become epidemiologically relevant if infection-prevention measures are applied inconsistently.

## Figures and Tables

**Table 1:** Characteristics of clinical *Candida* isolates and associated patients stratified by sample categorisation.

Sample Categorisation	*Candida* spp. Isolates, n	Patients, n *	Sex, Female/Male, n (%)	Most Common Sample Origin, n (%)	Total Most Common Candida Isolates, n (%) **	Species-Resolved Most Common Candida Isolates, n (%) Among Species-Resolved Isolates **
Invasive samples	8948	4042	1491 (36.9)/2551 (63.1)	Lower respiratory tract: 3341 (37.3); Normally sterile intra-abdominal specimens: 1908 (21.3); Blood cultures: 1444 (16.1); Sterile tissue: 980 (11.0); Other: 1275 (14.3)	*C. albicans*: 4698 (52.5); *N. glabratus*: 1831 (20.5); *C. tropicalis*: 495 (5.5); *P. kudriavzevii*: 399 (4.5); *C. dubliniensis*: 395 (4.4); Other: 1130 (12.6)	*C. albicans*: 4698 (54.7); *N. glabratus*: 1831 (21.3); *C. tropicalis*: 495 (5.8); *P. kudriavzevii*: 399 (4.6); *C. dubliniensis*: 395 (4.6); Other: 766 (9.0)
Non-invasive samples	41,709	23,299	15,960 (68.5)/7336 (31.5)	Upper respiratory tract: 13,486 (32.3); Urine: 13,099 (31.4); Vaginal swabs: 11,175 (26.8); Superficial skin swabs: 1630 (3.9); Other: 2319 (5.6)	*Candida* spp.: 33,648 (80.7); *C. albicans*: 4629 (11.1); *N. glabratus*: 1222 (2.9); *C. parapsilosis*: 459 (1.1); *P. kudriavzevii*: 424 (1.0); Other: 1327 (3.2)	*C. albicans*: 4629 (57.4); *N. glabratus*: 1222 (15.2); *C. parapsilosis*: 459 (5.7); *P. kudriavzevii*: 424 (5.3); *C. tropicalis*: 405 (5.0); Other: 922 (11.4)
Non-classifiable samples	3145	2199	923 (42.0)/1276 (58.0)	Unknown sample origin	*Candida* spp.: 1226 (39.0); *C. albicans*: 1092 (34.7); *N. glabratus*: 261 (8.3); *C. parapsilosis*: 150 (4.8); *P. kudriavzevii*: 123 (3.9); Other: 293 (9.3)	*C. albicans*: 1092 (56.9); *N. glabratus*: 261 (13.6); *C. parapsilosis*: 150 (7.8); *P. kudriavzevii*: 123 (6.4); *C. tropicalis*: 107 (5.6); Other: 186 (9.7)

* Patient counts are non-mutually exclusive across specimen groups because some patients contributed samples to multiple sample categorisation groups. ** Results obtained during the 42-day intensified species-level identification period were excluded.

## Data Availability

The original contributions presented in this study are included in the article. WGS data for the *C. auris* isolate CAU18 are available under NCBI BioSample SAMN55402253. Further inquiries can be directed to the corresponding author.

## Abbreviations

The following abbreviations are used in this manuscript:

MALDI-TOF MS	Matrix-assisted laser desorption time-of-flight mass-spectrometry
WGS	Whole-genome sequencing
MIC	Minimum inhibitory concentration
